# Posting Behaviour Patterns in an Online Smoking Cessation Social Network: Implications for Intervention Design and Development

**DOI:** 10.1371/journal.pone.0106603

**Published:** 2014-09-05

**Authors:** Benjamin Healey, Janet Hoek, Richard Edwards

**Affiliations:** 1 Department of Marketing, University of Otago, Dunedin, New Zealand; 2 Department of Public Health, University of Otago, Wellington, New Zealand; University College London, United Kingdom

## Abstract

**Objectives:**

Online Cessation Support Networks (OCSNs) are associated with increased quit success rates, but few studies have examined their use over time. We identified usage patterns in New Zealand's largest OCSN over two years and explored implications for OCSN intervention design and evaluation.

**Methods:**

We analysed metadata relating to 133,096 OCSN interactions during 2011 and 2012. Metrics covered aggregate network activity, user posting activity and longevity, and between-user commenting. Binary logistic regression models were estimated to investigate the feasibility of predicting low user engagement using early interaction data.

**Results:**

Repeating periodic peaks and troughs in aggregate activity related not only to seasonality (e.g., New Year), but also to day of the week. Out of 2,062 unique users, 69 *Highly Engaged Users* (180+ interactions each) contributed 69% of all OCSN interactions in 2012 compared to 1.3% contributed by 864 *Minimally Engaged Users* (< = 2 items each). The proportion of *Highly Engaged Users* increased with network growth between 2011 and 2012 (with marginal significance), but the proportion of *Minimally Engaged Users* did not decline substantively. First week interaction data enabled identification of *Minimally Engaged Users* with high specificity and sensitivity (AUROC  = 0.94).

**Implications:**

Results suggest future research should develop and test interventions that promote activity, and hence cessation support, amongst specific user groups or at key time points. For example, early usage information could help identify *Minimally Engaged Users* for tests of targeted messaging designed to improve their integration into, or re-engagement with, the OCSN. Furthermore, although we observed strong growth over time on varied metrics including posts and comments, this change did not coincide with large gains in first-time user persistence. Researchers assessing intervention effects should therefore examine multiple measures when evaluating changes in network dynamics over time.

## Introduction

Smoking continues to cause more deaths than any other preventable risk factor. [1] Policy interventions such as excise tax increases, [2], [3] marketing restrictions, [4] and more extensive smokefree environments, [5] have decreased smoking prevalence. Nevertheless, even countries with progressive tobacco control policies still report smoking prevalence of between 15% and 20%. [6], [7] Large, rapid increases in smoking cessation are necessary to achieve the ‘endgame’ objective of very low smoking prevalence (<5%) proposed in countries such as Finland, New Zealand, Scotland and Ireland. [8], [9]

Many smoking cessation interventions have limited effectiveness; [10] however, improved quit rates observed among smokers who have greater social support suggests interventions to enhance social support may increase quit success. [11] Models of contagion, originally developed to predict the diffusion of infectious diseases, are now being applied to analyse how diverse behaviours and conditions, including the spread of obesity, happiness, and smoking initiation, spread through social networks. [12]–[14] These models recognise that individual-level characteristics do not fully explain the uptake or extinction of risk behaviours, such as smoking, which institutional structures and cultural and social networks also influence. [15]–[17] Evidence that smoking cessation, like smoking initiation, may spread across networks independently of other factors, such as homophily [18] or policy constraints, suggests online cessation support networks (OCSNs) may not only support individual quit attempts but could also promote diffusion of smokefree behaviours.

Several factors have stimulated interest in smokers' digital social contexts and the role these may play in promoting and supporting cessation. These include growth in the reach and influence of Quitlines, many of which have associated OCSNs, and the increasing incorporation of online communities into daily activity. [19], [20] Observational evidence indicates that engagement with an OCSN is associated with higher smoking abstinence rates within web-assisted tobacco interventions. [21], [22] Moreover, recent studies document relationships between exposure to online cessation websites, cessation and successful abstinence. As participation increases, so too do reported abstinence rates, [23], [24] and the more messages posted, the greater the number of days reported as smoke-free. [23]

Higher OCSN activity is thought to promote stronger connections and provide reinforcement, particularly at times of stress, thus reducing quitters' propensity to disengage or lapse. [23] However, evidence is still relatively sparse and positive associations with quitting may result from self-selection. Nevertheless, while difficult to refute without data from suitably designed controlled trials using real-world OCSNs, the latter seems unlikely since it would require that none of the behavioural diffusion effects found in offline studies, [12], [16] or benefits of intervention tailoring apparent in online studies, [25] translate to OCSNs.

Given the current evidence, OCSN promoters thus have two objectives: first, they need to increase OCSN uptake so networks include a higher proportion of quitters. Second, they need to increase members' engagement activity and involvement in reciprocal relationships so a higher proportion of users receive support and, over time, are able to encourage others. Addressing the first question could reduce self-selection bias while attending to the second could ensure a higher proportion of members experience the benefits of greater activity. To approach both questions effectively, we require more detailed knowledge of existing usage behaviours over time.

Because OCSNs have wide reach in countries with high internet access penetration, continuous availability, and low marginal cost, they are potentially a highly cost-effective means to provide smoking cessation support and increase quit rates. It may be possible to improve their efficacy through interventions to provide better support to current users and maintain interactions by those currently likely to desist engagement very quickly. Developing such interventions should be informed by research that documents patterns of interaction among OCSN users. To date, however, few studies have examined aggregate network behaviour over time or how OCSN users interact within their community. [26]–[29]

Studies of North American OCSNs have highlighted the need for cross-disciplinary research to advance theory on how social networks influence smoking-cessation behaviour. [27] Other benefits of bringing varied perspectives to bear on how OCSNs do and could operate include better understanding of network formation, integration, retention and stability; creation of data-driven insights to inform the design and refinement of interventions; and better knowledge of different user populations within networks, particularly with respect to the degree and determinants of engagement. [27], [28]

Prior research suggests that individual usage of many online networks, including OCSNs, follows a skewed distribution with a few highly active users contributing a significant portion of content and a large tail of users who engage with the network only fleetingly. [26], [30], [31] Efforts to specify and identify such key groups will be important if they are to be targeted and incorporated into the OCSN design and development. Further, some evidence suggests that, within multi-faceted interventions such as national Quitlines, only a small fraction of registrants use the OCSN component. [21] These findings highlight the potential to increase both the number of OCSN users and the extent to which current fleeting users interact with the OCSN.

We analysed patterns in aggregate and individual engagement with a New Zealand (NZ) based OCSN with a view to informing future interventions that aim to stimulate or support OCSN interactions. Specifically, we aimed to:

1. identify patterns in aggregate posting behaviour over time;
2. explore whether network growth alters user engagement; and
3. examine the feasibility of identifying low-engagement users early in their cessation attempt.

## Data, Methods and Ethics

### The *QuitBlogs* intervention

In NZ, as in many other countries, the predominant OCSN (called the *QuitBlogs*) is operated by the national *Quitline* as a free service alongside telephone counselling, general online and text-based cessation advice, a ‘quit planning’ tool that enables users to set quit dates and note their reasons for quitting or triggers for cravings, and access to subsidised nicotine replacement therapy (NRT).

The *QuitBlogs* service was first offered in July 2006 with limited functionality allowing users to make public journal-style posts about their cessation journey. Commenting on other users' posts was only possible by creating a new journal entry and explicitly mentioning others' entries. Threaded commenting was added in August 2010 along with the functionality to ‘subscribe’ to updates from selected other users. In August 2013, ‘badge’ functionality was added, with users receiving badges against their username for achievements such as making 20 posts, ordering NRT, or completing a full quit programme (the latter being a three-month plus smoking cessation support programme comprising formulation of a quit plan and at least four follow-up support contacts).

The *QuitBlogs* do not allow for private messaging between users and posts are moderated by *Quitline* staff to ensure any offensive material is removed from the network. Only one staff account exists to comment on the network. This account answers specific user questions about NRT use, cravings, withdrawal or the *Quitline* services and does not attempt to promote engagement with the OCSN or between users.

Since launching, the number of interactions per year (posts or comments) on the *QuitBlogs* has grown from 805 in 2006/7 to over 93,000 in 2012/13 (up 61% from 2011/12). [32] Nevertheless, only approximately 15% of those attempting to quit using *Quitline* support read or interact on the *QuitBlogs* within a month of initiating a cessation attempt. [21]

### Data collection and selection

Using open-source software, we extracted the text and metadata (date, user ID, post ID, related post ID) relating to all posts or comments from the web pages publically available at http://www.quit.org.nz/blog/. Specifically, we developed a web crawler using Python 2.7 [33] and Scrapy 0.16.4 [34] to extract post and comment data along with related public profile information for each user; these data were stored in a MySQL [35] database created for the project. The extract covered posts or comments made from the service's beginning through to February 2013 and we used the full historic dataset to determine each user's first date of activity. However, our analyses focus on data relating to items from 01/01/2011 to 31/12/2012 (i.e., the analysis period) as these two years represent a period in which functionality did not change. Readers interested in viewing the *QuitBlogs* as they appeared during the analysis period are directed to an internet archive snapshot of the main blog page from July 2012 at http://goo.gl/VKnBrw.

Of the 134,782 posts or comments extracted and falling within the analysis period, 549 were identified as duplicates and excluded from further analysis. Duplicate identification involved calculating a SHA (Secure Hash Algorithm) hash of the text for each item within a thread using MySQL's SHA function. This approach created a small (160 bit) ‘digital fingerprint’ for each item and reduced the processing required to compare comments with one another, with extremely low risk of collision (i.e., different comments producing the same fingerprint).

We identified duplicates by selecting those for which the same hash corresponded to two or more items. Of the duplicates excluded, 119 related to one user whose account had been replicated due to a technical error. The remainder related to instances where users appear to have accidentally posted a comment multiple times within quick succession.

The *Quitline* staff account made 1,137 comments (excluding three duplicates) over the analysis period; these comments were also excluded from further analysis. After these exclusions, 133,096 items remained and represent the complete set of on-network interactions between *QuitBlogs* users over the analysis period. However, the data do not capture passive usage, such as *Lurking* (browsing by registered users who make no interactions or posts within the OCSN).

### Ethics statement

All data analysed were in the public domain (i.e., accessible publically on the *QuitBlogs* website) and our analysis was conducted with the permission of the *Quitline*. Since the data were publically available, the university staff member with delegated authority for ethics review for the project advised formal assessment was not required.

### Network user group definition

Researchers have explored levels of user activity using cluster analyses, [36] ‘top 100’ thresholds, [28] and network tie analysis combined with usage thresholds. [26] Using these methods, network participants have been classified into groups displaying high and low levels of network activity. The two main approaches to defining user activity categories are relative (e.g., top and bottom percentiles) and absolute (e.g., number of posts above or below a defined cut-point). Although arguably just as arbitrary as relative thresholds, absolute thresholds are more effective for examining user behaviours over time because they provide a consistent comparative benchmark. For instance, a comparison of top 1% users in period A to those in period B would show no change in membership, even if all users doubled their posting rates. Yet, group membership would change if the network grew in size but posting rates remained the same. The same is not true for an absolute threshold based on number of posts made within a period.

From a practical standpoint, absolute thresholds are also easier to implement in OCSN interventions. For example, an intervention aiming to reward users with a badge for their activity, or send a motivational email message to those who have not been very active, requires the development of automated systems to track usage and trigger actions. Absolute usage thresholds are simpler to develop than percentile or cluster-based thresholds because they do not require processing over the entire network to calculate. Furthermore, such thresholds are likely to be easier to understand and monitor by OSCN staff and clearer for users (who may be motivated to reach a certain activity achievement).

Since prior research on OCSNs indicated usage would follow a highly skewed usage distribution, and given the general scope of this study and the relatively simple functionality of the *QuitBlogs* network we investigated, we divided users into broad groups based on high and low absolute thresholds of posting behaviour for this analysis.

**Figure 1:**
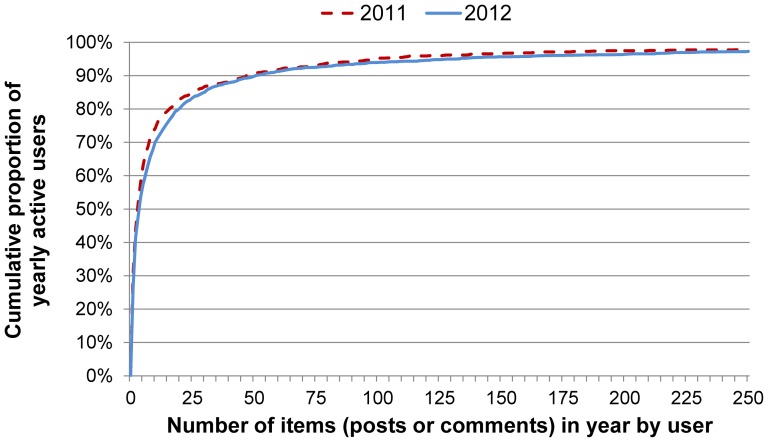
Cumulative distribution of user activity.

Specifically, we defined Highly Engaged Users (HEUs) as those contributing 180 or more posts or comments (items) within any three month period during a calendar year. This threshold covers users making sustained contributions to the network over an extended period (i.e., an average of two or more items per day over 90 days) and relates to the top end of the OCSN posting distribution (see Figure 1), although it may have excluded a small number of HEUs who commenced activity in the last two months of the year. We chose the rolling three month period along with the posting threshold to ensure that HEUs were not excluded simply because they commenced after the beginning of the year. Selection of a threshold corresponding to a percentile of posters in one year would have disadvantaged users starting later, who would be less likely to cross the threshold.

We defined Minimally Engaged Users (MEUs) as those contributing no more than two items within any three month period during a year. This threshold relates to the bottom end of the OSCN posting distribution and includes users who may have been fleetingly engaged in multiple ‘spells’ during the year (e.g., two posts in April and another two in August). The overall calendar year observational period was selected because it covers a full seasonal cycle and enabled comparisons between extended periods of time (i.e., 2011 and 2012).

In summary, we classify users according to the number of items they contribute to the network (posts or comments) within a defined activity window (three months, rolling).

### Analyses performed

We analysed aggregate interactions (all, first and repeat posts, and comments on posts) in 2011 and 2012 to identify recurring patterns and changes over time as the network grew. Day of week patterns in posting activity were modelled using generalised estimating equations (Poisson error distribution with identity link function). [37] The dependent variable was ‘total daily posts’, with ‘day of week’ the independent variable and clusters specified at the weekly level (to allow for non-independence of posting levels within any given week). Weeks in which a public holiday or World Smokefree Day occurred on a normal business day (i.e., Monday to Friday) were excluded from analysis, since these repeating events were known to cause large deviations from normal daily posting patterns.

We also examined how user engagement and persistence changed between 2011 and 2012, as measured by HEU and MEU group size and contribution. These analyses provide a view of typical OCSN structure and whether this varied over time.

Finally, we developed multivariable logistic regression models to assess whether early OCSN activity could be used to predict the subsequent MEU status of first-time users. Specifically, we developed two models using independent variables derived from ‘first day’ and ‘first week’ usage activity to predict MEU status in users who commenced activity between October 2011 and September 2012 (a full calendar year within the available observation period). MEU status (the independent binary variable) was determined using interaction information for 90 days after each user's start date (i.e., through to the end of 2012 for those commencing at the end of September 2012). We specified these models using a random 70% sample of users from that period and assessed them for accuracy using the remaining 30% hold-out group. Each model resulted in a score between zero and one for each individual in the hold-out group, relating to the estimated probability the user was a MEU. For model assessment, we considered any score above 0.5 to be a prediction that the user was a MEU. More details on the models are presented in the results section.

Statistical analysis was performed using R version 3.0.2 [38] and the RStudio development environment. [39] Figures presented in the results section in square brackets alongside the ± symbol represent 95% error margins. Error margins for the interaction contribution percentages for HEUs and MEUs were calculated using bootstrap re-sampling of users with the R *boot* package. [40]

## Results

### Patterns in aggregate posting behaviour over time

Of the 133,096 items analysed, 18,579 (14%) were blog posts, with the remainder being comments in response to those posts. Approximately 16% (2,915) of the blog posts were ‘first-time’ posts made by users who had not previously posted to the *QuitBlogs*; the other 84% were repeat posts. As Table 1 shows, growth occurred between 2011 and 2012 for first-time blog posts (up 46%), all blog posts (up 84%), and user-generated comments (up 118%). The average number of blog posts per user grew 24% from 2011 to 2012 [p = 0.01, difference between means] and comments per user grew 47% [p = 0.09, difference between means].

**Table 1:** Network user and posting metrics.

	Total	2011		2012		2011–12 Increase
All posts	18,579	6,536		12,043		84%
All comments	114,517	36,007		78,510		118%
First time posts	2,915	1,185		1,730		46%
Unique users	3,448	1,386		2,062		49%
Mean posts per user	5.4	4.7	[±0.6]	5.8	[±0.6]	24%
IQR		1–4		1–5		
Mean comments per user	33.2	26.0	[±9.2]	38.1	[±10.7]	47%
IQR		0–7		1–9		

Note: IQR =  Interquartile range. Figures in square brackets represent 95% error margins for the mean.

**Figure 2:**
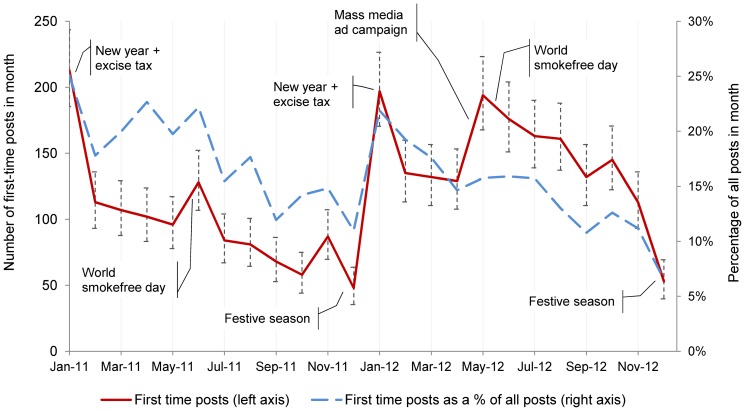
Patterns in aggregate first-time posting behaviour by month. Note: Error bars represent Poisson 95% confidence intervals for counts of first-time posts in each month.

New user activity spiked and troughed in broadly repeating patterns (see Figure 2). In both years, first time posts rose markedly in January (corresponding to New Year and annual increases in tobacco excise tax) and then declined until May, where there was an increase, possibly associated with World Smokefree Day. Activity then declined through to a low point in December, corresponding to Christmas-related festivities and holidays. In 2012, there was a large increase in activity during May which likely corresponded with the April launch of a television commercial campaign promoting the online *Quitline* services.

**Figure 3:**
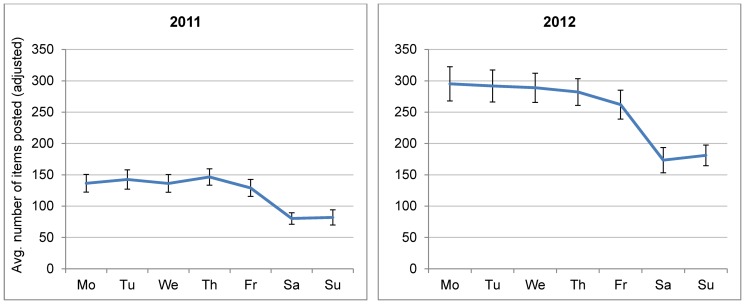
Mean items posted per week day, adjusted for weekly variation. Note: Error bars represent 95% confidence intervals for estimated mean number of posts.

There also appeared to be persistent day-of-week patterns in aggregate posting behaviour. Figure 3 presents the average number of items posted (i.e., blog posts or comments) per weekday for 2011 and 2012, adjusted for weekly variation using generalised estimating equations. Activity was highest during the working week but consistently low on Fridays. Although average activity on Friday was not significantly lower than Monday in 2011, it was in 2012 (p<0.001) and the two years prior to the analysis period (2009: p<0.001, 2010: p<0.05, not presented in Figure 3). Thus, the Friday effect existed across multiple years. Activity levels also consistently fell sharply on the weekend (p<0.001 for Saturday and Sunday compared to Monday in each year).

### Network growth and user engagement

**Figure 4:**
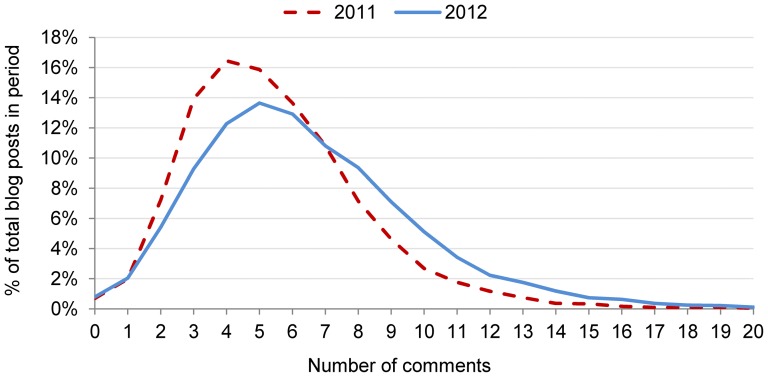
Comments per blog post.

There was a high level of interaction between OCSN users, with at least 90% of posts attracting three or more comments and only a small fraction (1%) receiving no comment at all (see Figure 4). The number of comments each blog post typically attracted increased from a mean of 5.5 [±0.1, IQR = 4–7] in 2011 to 6.5 [±0.1, IQR = 4–8] in 2012.

A small number of users contributed disproportionately to network activity. Specifically, 69 Highly Engaged Users (HEUs: 180+ comments or posts within any three month period, 3% [±1%] of users in 2012) contributed 69% [±8%] of all *QuitBlog* items during 2012. One user made over 6,000 comments (see Table 2). In 2011, the number of HEUs was lower; 33 HEUs (2% [±1%] of users) contributed 57% [±14%] of total interactions. While the total number of non-HEU network users grew 47%, the number of HEUs grew 109% between 2011 and 2012. The difference in percentage of HEU users between years (0.97%, 95%CI: −0.1% to 2.1%, p = 0.10) was marginally significant only at a relaxed 0.10 alpha level.

**Table 2:** Top 20 Highly Engaged Users in the cessation network during 2012.

User	Blog Posts	Comments	Distinct Days Active	Activity Span (Days)*
01	117	6,225	309	366
02	58	3,894	168	191
03	12	3,523	316	366
04	242	2,922	257	350
05	71	3,003	220	353
06	55	2,986	225	351
07	40	1,784	126	358
08	48	1,761	161	171
09	14	1,789	153	336
10	17	1,670	94	134
11	170	1,355	113	147
12	68	1,456	166	248
13	94	1,343	131	273
14	120	1,251	188	235
15	106	1,204	97	127
16	92	1,116	99	231
17	31	1,130	190	287
18	99	942	134	159
19	114	918	86	140
20	120	819	84	87

*Activity span measures the number of days between the user's first and last interaction during the year.

Around one third (12) of the HEUs in 2011 were also HEUs in 2012. This evidence of sustained activity suggests many HEUs remain prolific and persistent contributors to the network over at least the medium term. As shown in Table 2, most of the highest engaged users' contributions came through comments.

In contrast, 873 Minimally Engaged Users (MEUs: < = 2 interactions within any three month period, 42% [±2%] of users) contributed 1% [±0.4%] of interactions in 2012. In 2011, the network contained 615 such individuals (44% [±2%] of users) who contributed 2% [±0.6%] of total interactions. Of the 2011 MEUs, only 54 (9%) were also active in 2012. While the total number of non-MEU network users grew 54%, the number of MEUs grew at a slower rate (42%) between 2011 and 2012. The difference in percentage of MEU users between years (−2.0%, 95%CI: −5.4% to 1.3%, p = 0.24) was not significant.

### The feasibility of targeting low-engagement users

Interventions attempting to increase engagement amongst the large proportion of users who start but then quickly stop posting to the OCSN may need to identify whether or not a user is a MEU soon after they commence. Doing so will enable communication with users close to the point where their engagement is falling away. As outlined earlier, we therefore constructed logistic regression models to classify new users as MEUs or not using first-day and first-week posting information. Candidate independent first day and first week activity variables included: number of posts made by the user, number of comments made by the user (on their own or others' posts), percentage of total interactions by the user that were comments, distinct days of user activity (first week only), number of comments received on the user's posts, average number of other users commenting on the user's posts, and total post or comment word length (for posts or comments made by the user).

We retained four significant (p<.001) variables for the first-day activity model: number of posts made, number of comments made by the user (on their own or others' posts), percentage of total interactions by the user that were comments, and number of words posted by the user. Testing on the random cross-validation sample suggested a sensitivity of ∼75% (MEUs correctly identified) and specificity of ∼65% (35% false positive rate). The AUROC value was 0.77. [41] Other combinations of variables did not achieve higher accuracy.

In contrast, just two variables, number of posts made and number of comments made, were sufficient to generate accurate classification in the first-week activity model. This had a sensitivity of ∼95%, a relatively low false positive rate (∼15% of non-MEUs, specificity 85%), and an AUROC value of 0.94. The inclusion of other variables from the candidate set did not lead to substantive improvements in accuracy.

## Discussion

Approximately 15% of smokers initiating a quit attempt with the *Quitline* read or engage with the *QuitBlogs* within four weeks of initiating their attempt, [21] though many more may passively access the network as *Lurkers*. [30], [36] Nevertheless, the number of new *QuitBlogs* users in a given period represents a relatively small subset of all new *Quitline* registrants and substantial room may exist for growth in user numbers as well as active participation frequency in the *QuitBlogs* OCSN.

Efforts to increase participation and maximise the effectiveness of the *QuitBlogs* or other OCSNs require an understanding of the factors that influence network structure and interactions. This study contributes new insights relevant to three key OCSN intervention design factors: timing, user targeting, and evaluative benchmarking.

### Implications for intervention timing

Repeat patterns in day-of-week activity along with cyclic seasonal peaks and troughs in aggregate behaviour suggest opportunities to design and empirically test interventions involving proactively scheduled direct messages or prompts (e.g., via email) to current or potential OCSN users. A recent randomized trial of an education-style online cessation intervention using pre-prepared content with some tailoring to user characteristics found that basic weekly reminder prompts increased engagement, [42] but did not translate to cessation success rates above those for the intervention as a whole. [43] These findings suggest future research should examine whether contextualised (rather than fixed-schedule) message timing can also improve engagement. Future research could also explore whether improved engagement with dynamic and potentially self-reinforcing interventions such as OCSNs is more effective than engagement with a fixed-content intervention.

Social network based interventions could aim to increase network engagement, and thus quit support, by corresponding with expected changes in network dynamics or addressing anticipated needs in specific user groups. For example, tailored direct email messages in December (a trough period, with few first-time posts) may prompt existing OCSN users to increase network interactions which, in turn, could reduce the increased relapse risk possible during the festive season. Direct communications in January, a time when New Year's resolutions may prompt initial postings, could focus on facilitating new member integration. Although we highlight ideas for messaging here, other interventions that might be tested and compared include seasonal within-network competitions, awards, or public acknowledgements (e.g., badges).

A recent international study of cessation search trends on Google found the same day-of-week patterns we identified, with higher volumes during in the work week, dipping on Fridays and dropping sharply on weekends. [44] It also occurred for terms relating to healthy behaviours spanning multiple countries. [45] This consistent behavioural pattern may signal variations in relapse risk amongst quitters that warrant further exploration and, potentially, targeted interventions using complementary media. For instance, lower Friday and weekend activity could reflect increased offline social activities, in which case other media might provide more effective support, or OCSN avoidance due to lapses, which would suggest reactivation messages are appropriate.

Further research is needed to assess whether these peaks and dips in activity respond to tailored messaging or more targeted delivery channels. Since the day-of-week pattern appears to persist across time, region, and source, improved knowledge of predictable time-bound variations in relapse risk or propensity to seek support could also inform scheduling of non-network interventions, such as mass-media cessation advertising.

### Opportunities for user involvement and targeting

Network engagement exhibited the expected asymmetric distribution in our study; most users posted infrequently and a very small group of Highly Engaged Users (HEUs) contributed the majority of items. Analyses of North American OCSNs reported similar findings. [26], [28], [29]

When considered together, our results suggest a network effect associated with growth: as more people used the OSCN, the proportion of HEUs appeared to increase (albeit with marginal significance) along with the typical number of comments associated with each post. Nevertheless, the proportion of those who engaged fleetingly decreased only slightly, if at all, and large numbers of users remained minimally active across both of the years examined. Replication studies are required to establish whether these patterns generalise to other OSCNs.

Many HEUs had extended longevity in the network; they contributed frequently and were connected to many other users across the engagement spectrum. If accessible, HEUs could have a key role in developing interventions that alter, or capitalise on, existing network dynamics. Their insights could help develop interventions, such as more specific messaging around known risk periods, and their support is likely to be critical to the success of new initiatives.

At the other end of the engagement spectrum, interventions that seek to better integrate or reactivate MEUs into the OCSN may have the potential to improve access to cessation support and generate concomitant improvements in network efficacy. Certainly, evidence from other domains suggests that tailored and appropriately timed interventions can be effective. For example, motivational emails and contacts improved student retention and retrieval in a distance education setting, [46], [47] while tailoring improved engagement, retention and behavioural outcomes in an online intervention promoting consumption of fruit and vegetables. [48]

A recent Cochrane review of online interventions for smoking cessation also suggests that tailored and interactive approaches are more likely to improve cessation success. [25] Nevertheless, research is required to establish whether intervention activities incorporating elements of timing and tailoring can be successfully devised and deployed in OCSNs to improve engagement.

Early usage information could enable targeted interventions aiming to increase the engagement of MEUs as our results show that the number of posts and comments users make in their first week is sufficient to accurately identify the vast majority of MEUs with high sensitivity and specificity. This reasoning may appear syllogistic (i.e., MEUs do not interact much, so should be identifiable within a week) but it is not necessarily the case that one week of activity should be sufficient to identify them accurately. For instance, identification would be difficult in a network where even more engaged users had a posting frequency of only once or twice per week.

Our findings are exploratory and require further research to examine other potential predictors of disengagement and explore whether optimal timeframes for accurately identifying MEUs exist. Implementation practicalities and socio-behavioural factors may affect the trade-off between timing and accuracy. For example, an intervention could run weekly, contacting MEUs on a Monday (since this appears to be the day that health behaviours regain salience following the weekend), based on their first-time activity levels from the prior week. Such an intervention might aim to establish accurate MEU identification using data from the first 72 hours of a user's activity or, where this is not possible, wait until at least one week of data is available before entering a user onto the contact list.

Interventions similar to those described above could also hold the potential to engage *Lurkers*, who likely make up the largest percentage of potential users. In addition, research could explore how passive engagement (reading rather than posting) relates to cessation outcomes and to patterns of overall engagement with the OCSN. Although more difficult to quantify, aspects of passive engagement can be captured using browsing paradata. [48] Currently, routine collection of paradata may not be widespread. More research is required to determine how best to collect and utilise this information in combination with active engagement data and follow-up cessation outcome records.

Selby et al's [29] analysis of first time users found many first posts were made by struggling recent quitters, while comments came mainly from those who had successfully quit for more than a month. [31] Many of these supporters are likely to have been HEUs, who appear to make the vast majority of their contribution to the network through comments rather than creating new threads. Together, our findings suggest that, rather than merely increasing the number of comments MEUs' posts receive, interventions could focus on comment style to improve the likelihood that postings stimulate a response and then a conversation, or even increased passive engagement with the intervention. Specifically, future research could test interventions developed in collaboration with HEUs to encourage first-time posters to interact with other users, while ‘reactivation’ messages to identified MEUs could aim to stimulate a return to network engagement.

### Metrics for benchmarking OCSN activity

We present several aggregate and individual-level network engagement metrics that enable monitoring of OCSN dynamics over time. In the OCSN examined, we observed growth on varied measures including new posts, comments, and HEUs, although this growth translated into only limited increases in persistence by first-time users. Since these metrics do not all necessarily move in the same direction, or at the same rate, researchers assessing intervention effects should examine multiple measures when evaluating changes over time.

### Strengths, limitations and directions for future research

This study is the first that we are aware of to report aggregate OCSN activity patterns over an extended timeframe, track changes in the size and contribution of high or low engagement users during network growth, and explore early detection of low engagement individuals using activity data. As such, our findings add important context to the limited number of other studies that have examined network structure or specific user groups at given points in time. [26]–[29] We found broad similarities in the asymmetric network structure of the OCSN we examined and in the North American OCSNs studied previously. [26], [28], [31] These commonalities, together with evidence that day-of-week posting patterns in our OCSN parallel those identified in recent international search engine studies, [44], [45] suggest our findings will have international relevance to OCSN intervention design.

Our reasoning assumes that improvements in OCSN engagement will translate into increased cessation success or longevity. However, we did not have access to cessation information for the users examined, although an evaluation of the *Quitblogs* users found higher self-reported levels of cessation. [21] We also did not have access to total current *Quitline* user registration information over time for our analysis. As such, our data do not allow us to explore the extent to which the seasonal ‘first posting’ patterns we present were due to general increases in *Quitline* registrations versus time-bound variation in propensity for *Quitline* users to become active on the *QuitBlogs*. Future intervention-based research should incorporate measures of cessation success and broader service registration for evaluative purposes.

The OCSN we examined was part of a multi-component cessation intervention that included other optional treatment components such as NRT and telephone counselling. Future studies on similar OCSNs might assess how engagement data from those components, where available, could be used to optimise OCSN engagement.

Furthermore, our metadata did not enable detailed analysis of interactions between users within threads (i.e., commenting on comments). Further research is also required to examine the extent and complexity of within-thread activity, and its behavioural implications. Given the potential importance of message tone in stimulating enduring engagement, we also recommend that research examine the qualitative nature of posts (e.g., tone, sentiment, style or topic) and the effect of tone on the engagement and quitting success of message recipients. Related to this, studies exploring the early identification of those users more likely to become HEUs may enable interventions to involve them even more productively in the network.

As noted in the methods section, we chose operational definitions for MEUs and HEUs based on absolute usage thresholds that were likely to be relevant and useful for intervention designers. However, these definitions were as arbitrary as any other that could have been chosen and the selection of different thresholds would have yielded results with a different context and operational focus. Additional work relating usage threshold definitions to observed cessation rates would be useful to inform optimal levels for intervention focus and evaluation.

Finally, we were unable to examine differences between users with different quit histories (i.e., whether they were making their first quit attempt, making a repeat attempt, or had been abstinent for some time). Other studies [26], [29] report differences in interactive behaviour between these user groups. Longitudinal research could therefore explore how groups with different cessation histories engage with OCSNs and respond to varied interventions.
